# Restricting Prey Dispersal Can Overestimate the Importance of Predation in Trophic Cascades

**DOI:** 10.1371/journal.pone.0055100

**Published:** 2013-02-07

**Authors:** Nathan R. Geraldi, Peter I. Macreadie

**Affiliations:** 1 Institute of Marine Sciences, University of North Carolina at Chapel Hill, Morehead City, North Carolina, United States of America; 2 School of the Environment, University of Technology, Sydney, New South Wales, Australia; Leibniz Center for Tropical Marine Ecology, Germany

## Abstract

Predators can affect prey populations and, via trophic cascades, predators can indirectly impact resource populations (2 trophic levels below the predator) through consumption of prey (density-mediated indirect effects; DMIEs) and by inducing predator-avoidance behavior in prey (trait-mediated indirect effects; TMIEs). Prey often employ multiple predator-avoidance behaviors, such as dispersal or reduced foraging activity, but estimates of TMIEs are usually on individual behaviors. We assessed direct and indirect predator effects in a mesocosm experiment using a marine food chain consisting of a predator (toadfish – *Opsanus tau*), prey (mud crab - *Panopeus herbstii*) and resource (ribbed mussel – *Geukensia demissa*). We measured dispersal and foraging activity of prey separately by manipulating both the presence and absence of the predator, and whether prey could or could not disperse into a predator-free area. Consumption of prey was 9 times greater when prey could not disperse, probably because mesocosm boundaries increased predator capture success. Although predator presence did not significantly affect the number of crabs that emigrated, the presence of a predator decreased resource consumption by prey, which resulted in fewer resources consumed for each prey that emigrated in the presence of a predator, and reduced the overall TMIE. When prey were unable to disperse, TMIEs on mussel survival were 3 times higher than the DMIEs. When prey were allowed to disperse, the TMIEs on resource survival increased to 11-times the DMIEs. We found that restricting the ability of prey to disperse, or focusing on only one predator-avoidance behavior, may be underestimating TMIEs. Our results indicate that the relative contribution of behavior and consumption in food chain dynamics will depend on which predator-avoidance behaviors are allowed to occur and measured.

## Introduction

The relative importance of predation and trophic cascades remains a central focus in community ecology [1]–[5]. Studies on trophic cascades originally focused primarily on the indirect effect of predators that are generated by their consumption of prey, but there is a growing number of studies that have found that predator-avoidance behavior of prey is also important to understanding food web dynamics and community structure [6]–[8]. For example, studies examining the cascading effects of predators on prey-resource dynamics have shown that resource persistence is influenced as much by these behavioral or trait-mediated indirect effects (TMIEs) as by predators consuming prey (density-mediated indirect effects; DMIEs) [7], [9], [10]. For instance, in grassy meadows, spiders feeding on grasshoppers had a similar positive effect on grasses as when spiders without working mandibles were present [11]. Hence, the inclusion of prey behavior in food web models is an important step towards developing a holistic understanding of ecological processes [12], [13].

TMIEs often result from more than one predator-avoidance behavior in nature [14]. For example, elk, under the risk of predation by wolves, increase vigilance time and decrease foraging [15], [16], which results in increasing willow heights [17], [18]. In addition, elk can also alter habitat selection in the presence of wolves and move away from their preferred resource in open grasslands to safer coniferous forests with lower-quality resources [19]. Because animals use multiple behaviors in response to predators in nature, studies measuring the relative importance between TMIEs and DMIEs should quantify multiple predator avoidance behaviors [14], [20]. Determining the relative importance of TMIEs and DMIEs is necessary in order to include behavior in food chain models that have until recently included only the effects of consumption. In a recent meta-analysis on the relative importance of prey behavior and predation in indirect interactions, 20 studies measured TMIEs and DMIEs [7]. Three different predator avoidance behaviors of prey were quantified in addition to prey mortality; reduced activity, changes in habitat, and immigration, but only one predator avoidance behavior was measured at a time in these studies ([7]; Table S1). This practice of measuring one predator-avoidance behavior at a time, according to Preisser et al. [7] behavior categories (these categories will be used throughout this paper), may lead to an incorrect assessment of indirect effects when compared to natural settings.

One of the most frequently studied predator avoidance behaviors in indirect effect studies is prey activity, but studies on prey activity that are conducted in mesocosms usually restrict the ability of prey to disperse to locations where the threat of a predator is diminished [11], [21]–[23]. Studies that measured the effect of reduced prey activity on indirect effects in Preisser et al. [7] had mesocosm boundaries that limited dispersing prey to distances that we estimate prey could move in less than a minute (Table S1). Restricting prey to an area that is small compared to the area that they use in nature (home range) inhibits the prey’s ability to disperse from predators. The ability of prey to move away from the threat of predation can depend on the density of predators and the distance at which prey are able to detect a predator. Even if mesocosm size does not alter the ability of prey to disperse, mesocosms boundaries can alter detection and capture of prey [24]. Furthermore, minor changes in predator-prey interactions can have major impacts on resources [7]. Thus, mesocosm experiments measuring the effects of predators on prey activity could overestimate DMIEs because prey are unable to disperse from the threat of predation and mesocosm boundaries increase predator capture success.

The indirect effect of prey dispersal on the resource has been tested in enclosure studies in streams [25]–[28] and grasslands [29]. These studies found that dispersal was more important than predation in determining local resource density, but they did not assess how resource survival was affected by predator consumption of prey and reductions in prey foraging because of difficulties in determining the number of prey eaten versus the number of prey that dispersed [30]–[32].

Our study system consisted of a tri-trophic food chain with toadfish (*Opsanus tau*; predator), mud crabs (*Panopeus herbstii*; prey), and ribbed mussels (*Geukensia demissa*; resource). Past experiments have been conducted with similar species in 1.7 m diameter mesocosms and found that toadfish indirectly benefit juvenile oysters or clams, and that the relative importance of TMIEs, resulting from reduced activity in the presence of a predator, was much greater than DMIEs [22], [23]. However, a study on the mobility of the same species of mud crab found that marked crabs released in the wild were not found within 5 m of the release point after 48 h [33]. Consequently, we designed an experiment that manipulated the presence and absence of a predator within mesocosms that either prevented or allowed prey, but not a predator, to disperse out of the mesocosm. The design allowed for the predator to affect the prey through consumption and behavior, including the density of prey via consumption and dispersal as well as the traits of prey via activity and dispersal. The importance of reduced activity of prey, prey dispersal, and predation of prey were each quantified to assess the indirect effects of the predator on the resource.

## Materials and Methods

### Ethics Statement

This experiment was conducted in accordance with the Public Health Service policy on Humane Care and Use of Laboratory Animals, the Amended Animal Welfare Act of 1985, and the regulations of the United States Department of Agriculture. The methods were approved by the University of North Carolina at Chapel Hill Institutional Animal Care and Use Committee (Application Number: 09-111.0-B).

### Quantifying Indirect Effects

Assessments of the importance of prey behavior in ecological processes must isolate behavioral effects from consumptive effects [6], [8], [34]. Experiments accomplished this by parsing different indirect effects via counting the numbers of the resource species eaten per day by prey in the absence (M) and presence (m) of a predator, the daily per-prey consumption of the resource in absence (C) and presence (c) of a predator, and the number of prey eaten by a predator (p; Table 1). The DMIEs were the amount of resources surviving because of prey mortality (c·p). The actual release (AR) was the difference between resources consumed by prey in the absence and presence of a predator (M-m). The activity resource release (AyR), or the amount of resources that were not eaten because prey reduce activity and foraging in the presence of a predator, was the difference between the AR and the DMIEs [22], when dispersal was prevented. Thus, if the change in the numbers of resources and prey are known, indirect effects can be estimated (Table 1). We took this construct one step further by calculating the dispersal resource release (DR), the positive effect of a predator on resource survival resulting from prey dispersal, by multiplying the per-prey consumption of resources by the number of prey that dispersed and then subtracting the number of resources not eaten because of dispersal in the presence (c·e) and absence (C·E) of a predator. The increase in resource survival resulting from reduced prey activity was then calculated in mesocosms that allowed prey dispersal (AyR = AR-DR-DMIE; Table 1).

**Table 1:** The variables and formulas used to calculate indirect effects (upper panel) and the experimental results (lower panel).

Mesocosm condition	Predator	No. crabseaten	No. crabs dispersed	Mussel consumption (mussels·d^−1^)	Standardized mussel consumption (mussels· crab^−1^ ·d^−1^)	Predation resource release† (DMIE, mussels ·d^−1^)	Actual resource release (mussels·d^−1^)	Dispersal resource release‡ (mussels ·d^−1^)	Activity resource release§ (mussels·d^−1^)	Dispersal &activity resourcerelease|| (TMIE,mussels·d^−1^)	Indirect effects attributable to TMIE (%)
Closed	No			M	C	DMIE = p·c	AR = M-m		AyR = AR-DMIE	TMIE = AyR	(AR-DMIE)/AR
	Yes	p		m	c						
Open	No		E	M	C	DMIE = p·c	AR = M-m	DR = (c·e)-(C·E)	AyR = AR-DMIE-DR	TMIE = AyR+DR	(AR-DMIE)/AR
	Yes	p	e	m	c						
											
Closed	No			8.70±1.82	1.82±0.33	1.31±0.80	5.50±1.74	(0)	4.19±2.06	4.19±2.06	76%
	Yes	1.33±0.42		3.19±1.32	0.80±0.36						
Open	No		1.33±0.21	10.00±2.70	2.22±0.60	0.46±0.46	5.75±2.97	−2.21±0.70	7.58±3.46	5.37±3.13	92%
	Yes	0.17±0.17	1.33±0.42	4.25±1.83	0.97±0.43						

Lower case letters denote presence of predator and upper case denotes absence of predator. Estimation of the resource release resulting from predators consuming prey (DMIE), the dispersal of prey (DR), the reduction in prey activity induced by a predator (AyR), and the percent contribution of predator-avoidance behaviors in indirect effects.*Note:* Mean ± standard error.

† Predation resource release is the rate at which mussels were not eaten by mud crabs because the crabs were consumed by toadfish.

‡ Activity resource release is the rate at which mussels were not eaten as a result of the changes in crab behavior in the presence of toadfish.

§ Dispersal resource release is the rate at which mussels were not eaten as a result of the movement of crabs from the mesocosm to the sanctuary.

|| Dispersal and activity resource release is the rate at which mussels were not eaten as a result of all the predator-avoidance behaviors measured.

Our mesocosm design was based on a combination of past research on prey dispersal, which has primarily been measured in stream mesocosms (referred to as emigration in those studies), and research on changes in prey activity, which has primarily been conducted in closed mesocosms mimicking marine or terrestrial environments. Crab movement out of the mesocosm was considered dispersal and not refuge-seeking behavior because we refer to dispersal as the movement out of a risky environment while refuge seeking behavior is hiding within a risky environment. Refuge seeking is a reduction in crab activity when crabs hide deeper within the oyster shell to escape predation [23].

### Experimental Setup

Experiments were conducted in 21 m^2^ outdoor cement ponds (7×3 m) at the University of North Carolina’s Institute of Marine Sciences (Morehead City, NC, USA). Animals were collected by hand or trap in Bogue Sound under a North Carolina Division of Marine Fisheries research collection permit to the Institute of Marine Sciences and held in flow-through tanks supplied with raw seawater (1 Ls^−1^). Toadfish were fed chunks of frozen fish and crabs were fed mussels (>1 cm shell height) *ad libitum* every 2 days before experiments started.

**Figure 1:**
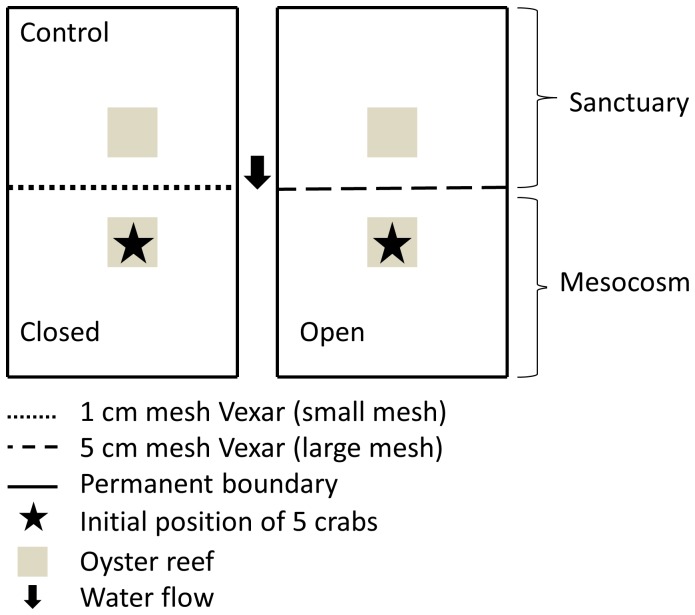
The experimental setup of the study showing open and closed mesocosms and the initial placement of mud crabs.

The experimental design consisted of 2 crossed factors: predator (present or absent) and mesocosm design (open - prey could leave the mesocosm or closed - prey could not leave mesocosm). The 2 mesocosm designs were created by dividing each cement pond in half with one of 2 alternate sizes of Vexar mesh, one of which allowed crab dispersal (open –5 cm mesh), while the other did not (closed –1 cm mesh). Depending on the mesh size crabs could move out of the mesocosm (3.5×3 m) into a predator-free sanctuary (other side of the cement pond; Fig. 1). The sanctuary in the closed treatment was used as a control to measure mussel mortality not attributable to crab consumption.

Oyster habitat was created by adding cleaned adult oyster shells (37.9 L bucket full of shells) to each mesocosm and sanctuary, and spreading it out to cover a 0.56 m^2^ area. The oyster shells were approximately 15 cm deep. The oyster habitat was placed 0.5 m from the mesh barrier so that oyster habitat in open mesocosms was 1 m apart from oyster habitat in sanctuaries (Fig. 1). The size of the oyster habitat and the distance between patches are commonly found in natural oyster reefs (Fig. S1; [35], [36]). Eight oyster shells each had 7 mussels (resource) attached to them and were haphazardly placed within the oyster habitat. Mussels are commonly found in interstitial spaces in oyster reefs and are eaten by mud crabs [37]. Mussel shell height ranged from 8 to 24 mm (17±0.04, mean ± standard error, n = 60). Mussels were placed on the shell 24 hours before the beginning of the trial and they naturally attached to the oyster shell via their byssal threads.

Five mud crabs (range = 10–39 mm carapace width, mean ± SE = 26±0.4 mm, n = 119) were placed in the oyster habitat in each mesocosm (Fig. 1). Crab density within the oyster habitat in mesocosms (8.9 crabs m^−2^ of oyster habitat) was selected from the lower end of the natural range of crab density for individuals with 20–40 mm carapace widths within intertidal oyster reefs in South Carolina (4–20 m^−2^; [38]) to reduce density-dependent movement and interference competition. After crabs had acclimated for 30 min in the mesocosm, a single adult toadfish (range = 230–320 mm total length, mean ± SE = 278±1.0 mm, n = 8) was added to the mesocosm of the predator-present treatments. Each trial of the experiment consisted of a single replicate of each of the 4 treatments (predator present or absent crossed with mesocosm open or closed). Replication was gained through successive trials (n = 6) and treatment was haphazardly assigned to mesocosms before each trial. Trials were run from July to Sept in 2009. Trial time was based on keeping resources above 50% to minimize crab dispersal resulting from resource depletion and to minimize a decrease in prey feeding rate because of resource depletion [39], which was measured in pilot trials and took 2–3 days. Mesocosms were completely drained of seawater at the end of each trial, which required approximately 15 min, to allow crabs and mussels to be accurately counted. Individual animals were only used once and released after each trial.

Observations of crab location were conducted 3–4 times during each trial. The observations were conducted from 8 am –8 pm. Each mesocosm and sanctuary was searched for 1 min and the location of each visible crab was recorded. The locations of crabs were grouped into 4 categories: within oyster habitat, closer than 5 cm to mesocosm walls, in mesocosm corners, or in the open (between oyster reef and mesocosm boundaries).

### Experimental Design Justification and Caveats

The experimental design allowed us to quantify the number of prey that dispersed out of the mesocosm in the absence and presence of a predator. The use of a mesh barrier, which was necessary to allow mud crabs to disperse while still measuring changes in prey and resource abundance resulting from consumption and dispersal, may have caused experimental artifacts because it prevents predators from chasing prey into the sanctuary. An additional experiment was run to quantify prey mortality when the predator could or could not move into the sanctuary. The average number of prey eaten when the predators could move into the sanctuary was, on average, greater (0.83±0.17; mean ± standard error) than when they could not (0.33±0.21). However, these results do not provide a true test of any potential artifacts of the mesh barrier because allowing predators to move into the sanctuary results in the same mesocosm-wall restrictions as the closed mesocosm but with twice the foraging area, thereby confounding the comparison. Nevertheless, toadfish are ambush predators and their attack is characterized by a quick and sudden strike, and toadfish are sedentary and occupy dens (tin cans or piles of oyster shell) for 3 to 5 weeks [40]. Thus, it is unlikely that crab predation was affected by the mesh barrier.

Past studies calculate TMIEs [10], [41] by using a “risk” or “cue” treatment. Risk treatments usually consisted of a predator that is caged within mesocosms or water flowing through a tank containing a predator before flowing into the study mesocosm. We did not include a risk treatment because risk treatments can underestimate predator-avoidance behaviors because prey never have an opportunity to escape the predator [20] and the reduction in prey foraging resulting from predator presence is calculated from per prey consumption of resources when the predator can consume the prey. However, the risk treatment does keep prey density constant and removes any artifact resulting from crabs altering their feeding rates with changes in crab density. We acknowledge that this could bias our results if crabs increased feeding when crab density decreased from either predation or dispersal. But, the experiment was designed to minimize changes in density-dependent crab feeding rates and intraspecific interactions by using crab densities from the lower end of natural densities and with resources that were distributed throughout the oyster habitat.

### Statistical Analysis

Response variables were analyzed in factorial ANOVAs with mesocosm design (open or closed) and predator (present or absent) as fixed factors and trial (1–6) as a blocked factor. Dependent variables were: proportion crabs consumed (crabs eaten/{[initial # of crabs+final #of crabs]/2}), proportion crabs remaining in the mesocosm (final # of crabs/{initial # of crabs+# crabs eaten}) and percent mussels consumed in the mesocosm. ANOVAs were also run for results in the sanctuary (open mesocosms only) with predator (present or absent) in the mesocosm as a fixed factor and trial (1–6) as a blocked factor. Dependent variables for ANOVAs run with results from sanctuaries were: number of crabs in the sanctuary at the end of the trial, and percent mussels consumed in the sanctuary. All data were first tested for normality and homoscedasticity by the K-S normality test and the Levene’s test, respectively. Data passed both tests without transformation unless stated otherwise. A p-value <0.05 was used to determine significance. In addition, a p-value between 0.05 and 0.1 was considered marginally significant because the dependent variable may be ecologically significant. However, marginally significant results should be interpreted with caution because of the potential for Type 1 error.

Crab behavior was analyzed using a 3-way MANOVA with predator, mesocosm design and trial as independent factors. The numbers of crabs observed per trial along the sides, in corners, and in oyster habitat were the dependent variables. The number of times a crab was observed in each trial was divided by the average number of crabs present and the number of observations conducted during that trial to account for differences in the number of crabs and observations among trials. Only 1 crab was observed in a sanctuary and only observations in mesocosms were used in the observation analysis. A crab was never observed in the open so this category was not used in the analysis. To elucidate which observation category was driving the significant MANOVA results, separate three-way ANOVAs, with predator (fixed), mesocosm design (fixed), and trial (blocked) as independent factors, were run with each location category as the dependent variable.

### Indirect Effect Calculations

To determine the effect of mesocosm design, prey activity within the mesocosm, and prey dispersal on the relative strength of DMIEs and TMIEs, we used calculations similar to Grabowski [22]. Variables and equations are shown in Table 1; lower case variables indicate predator presence and upper case variables indicate predator absence [8]. The mean number of crabs eaten by a predator during a trial (p) was calculated for closed and open mesocosms. We determined the mean number of crabs that dispersed out of open mesocosms during a trial with (d) and without predators (D), as well as the per-prey rate of resource consumption for open and closed mesocosms with (c) and without (C) a predator. All calculations were carried out independently for open and closed mesocosms. The rate of resource consumption per prey was calculated by dividing the number of resources consumed per day by the average number of crabs present during the trial. The average number of crabs was calculated by dividing the initial plus the final number of crabs by 2.

DMIEs, or the number of mussels surviving because of predation of mud crabs, was calculated for predator treatments (p·c). Actual resource release (AR), or the number of mussels not eaten because of the presence of a predator, was calculated by subtracting the mussel consumption without and with a predator (M-m). Dispersal resource release (DR), or the number of mussels not eaten because of crab dispersal out of the mesocosm and away from the predator, was calculated by subtracting the number of mussels not consumed because of crab dispersal without a predator present (C·D) from the number of mussels not consumed because of crab dispersal in the presence of a predator (c·d) in the mesocosm. Dispersal resource release was only calculated for open mesocosms. Crab consumption rates of mussels in the presence and absence of predators was used to incorporate the difference in mussel survival when crabs disperse from areas with or without predators. Including this difference is more similar to the effect of a sedentary predator on resource survival in natural settings then assuming crabs consumption rates remain constant regardless of predator presence (i.e. DR = C*d-C*D).

The activity resource release (AyR), or the number of mussels not eaten because of mud crabs reducing activity in the presence of a predator, was calculated for closed mesocosms (AyR = AR-DMIE). The calculation for AyR in open mesocosms included the number of resources not eaten because of crab dispersal (AyR = AR+DR –DMIE; Table 1). TMIEs or the total indirect effects resulting from predator-avoidance behaviors were calculated for closed (AyR) and open mesocosms (AyR+DR). Finally, the relative magnitude of TMIEs compared to the total indirect effect of the predator on the resource was calculated for open and closed mesocosms by dividing TMIEs by the sum of indirect effects (DMIE+TMIE; Table 1).

The contribution of the DMIE can be calculated by subtracting the TMIEs from 1. Standard errors were not calculated for the indirect effect percentages because one trial in both AyR and DMIE calculations had a negative number, which resulted from more mussels being consumed in the presence of a predator for those trials. The negative number greatly skewed the calculations by reducing the mean even when transformations were conducted. Thus, the means of the resource release were used and error was not calculated.

## Results

**Figure 2:**
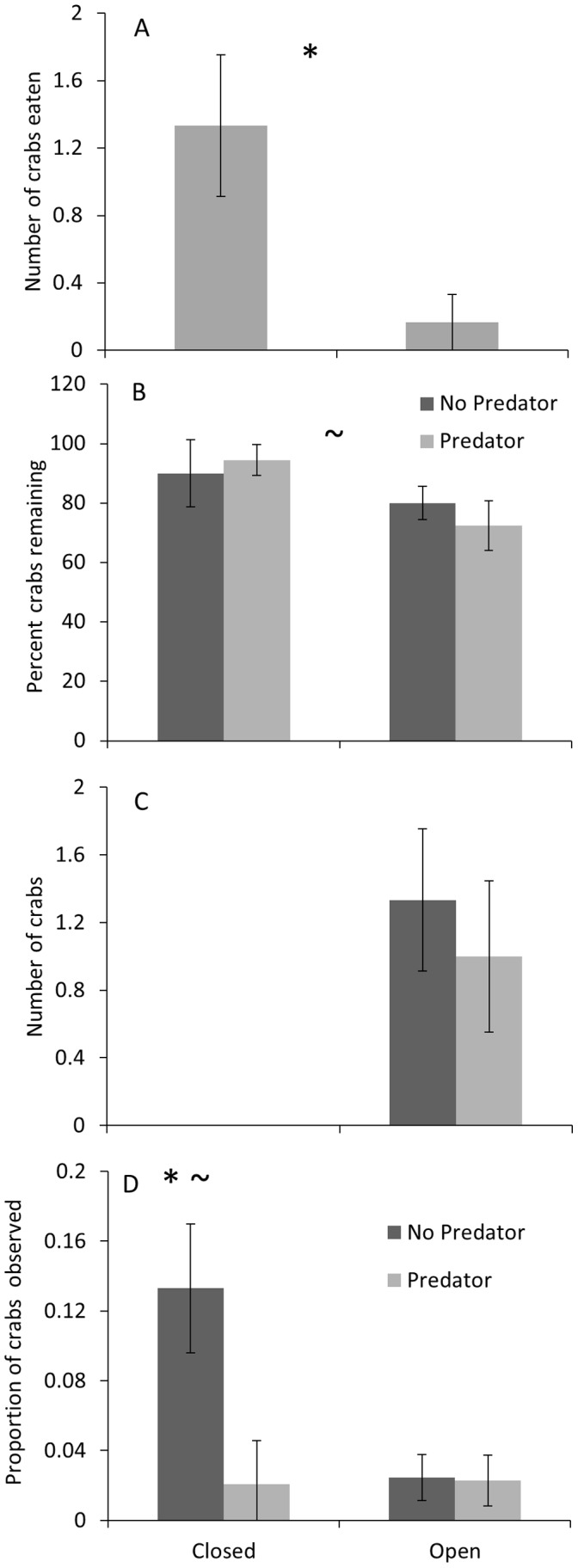
Summary of crab (prey) mortality, survival, and behavior. Results showing: (A) the mean proportion (±SE) of crabs eaten per trial by toadfish in open and closed mesocosms; (B) the mean proportion (±SE) of surviving mud crabs remaining in the mesocosms; (C) the mean number (±SE) of mud crabs that dispersed into sanctuaries; and (D) the proportion of crabs observed along the edges of mesocosms. The number of crab observations was standardized for both the number of observations per trial and by the average number of crabs. Significant effects (p<0.05) of mesocosm design are indicated by asterisks, and the significant effect of toadfish presence/absence is indicated by a tilde. All results are for crabs in original mesocosms, except for C.

The proportion of crabs consumed by toadfish was 8 times higher in closed (0.35±0.11 crabs per trial; mean ± standard error; n = 6 for all analyses) than in open mesocosms (0.041±0.041 crabs per trial; F_1, 11_ = 6.64, p = 0.030; Fig. 2 A; Table S2). Predator presence did not affect the proportion of surviving crabs remaining in mesocosms (final # of crabs/(initial # of crabs- # crabs eaten); F_1,15_ = 0.03, p = 0.857; Fig. 2 B; Table S3), but mesocosm design did affect the proportion of surviving crabs remaining in the mesocosm with more crabs remaining in the closed mesocosms, although only marginally significant (F_1,15_ = 3.66, p = 0.075; Fig. 2 B; Table S3). Thus, predator presence did not affect crab dispersal, but crabs did disperse when in open mesocosms. The closed mesocosms did not have all of the crabs remaining in the mesocosm because 3 crabs in no predator trials and 2 crabs in the predator trials managed to get under the small mesh barrier and moved into the control. This should not have affected our results because so few crabs escaped from closed mesocosms. Toadfish presence did not affect the number of mud crabs that were in the sanctuary at the end of the trial (F_1,5_ = 0.19, p = 0.679; Fig. 2 C; Table S4).

**Figure 3:**
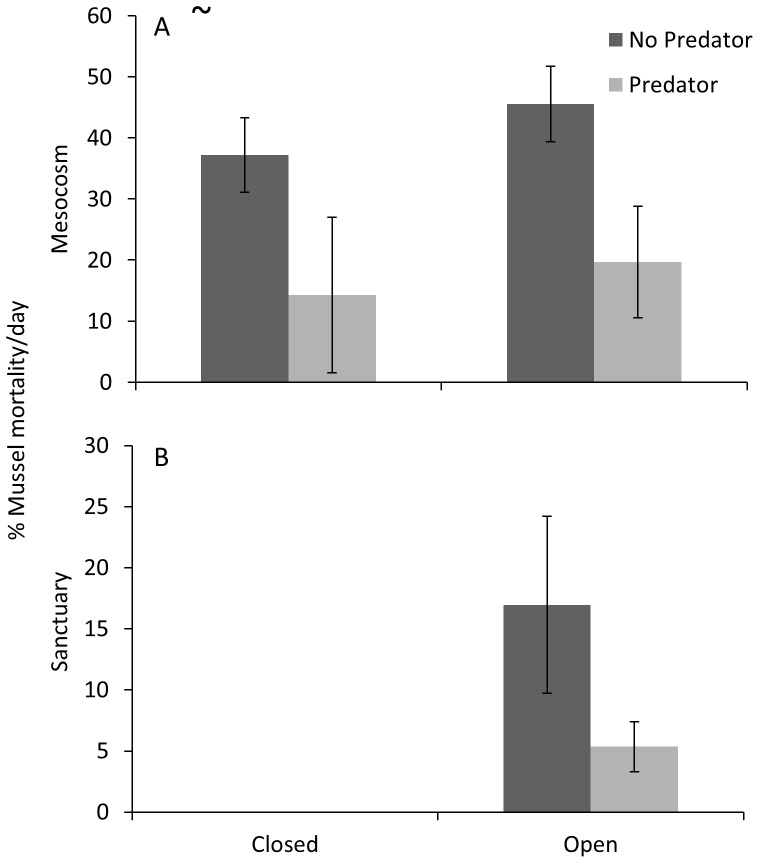
Summary of mussel (resource) mortality. Results showing: (A) the percent mortality of mussels per day in mesocosms and; (B) the percent mortality of mussels per day in sanctuaries. Significant effect (p<0.05) of toadfish presence/absence is indicated by a tilde. Toadfish presence/absence was marginally significant in the sanctuary (p = 0.092).

All mussel mortality was assumed to be from mud crab consumption because mussel mortality in the control sanctuary was negligible (0.6±0.25 mussels per trial) and toadfish did not eat mussels (N. Geraldi pers. obs.). Toadfish presence reduced mussel mortality by half (F_1,15_ = 11.38, p = 0.004), but there was no difference in mussel mortality between open and closed mesocosms (F_1,15_ = 0.50, p = 0.490; Fig. 3 A; Table S5). Percent mortality of mussels in the sanctuary was reduced from 17 to 5% when the mesocosm had a predator, which was marginally significant (F_1,5_ = 4.32, p = 0.092; Fig. 3 B; Table S6). Neither the trial factor nor the interaction term had an effect (p>0.10) for any of these statistical tests.

Although the ability to observe crabs was limited by variable water turbidity; observations of all treatments during trials were conducted 21 times (3–4 observations during each trial) and 33 crabs were observed during the entire experiment. The majority of crabs was observed along the edges of the mesocosms (20), and these crabs were moving in 75% of the observations. A total of 10 crabs was observed in the corners, and these corner crabs were inactive in 90% of the observations. Three crabs were observed in the oyster habitat. There was a significant interaction between predator and mesocosm type (F_1,20_ = 4.059, p = 0.023), and predator was marginally significant (F_1,20_ = 2.445, p = 0.097) when observations of crabs in corners, in oyster reef, and along edges were analyzed using a MANOVA (Table S7). The proportion of crabs was not normally distributed among the three dependent variables and crabs along edges did not have homogeneous variances. When transformation did not improve normality or heteroscedasticity, the variables were left untransformed. Non-parametric tests were not run because they cannot analyze mixed-effect models. Although variance tests are robust to non-normal data [42], caution should be taken in interpreting the ANOVA for proportion of crabs along edges because this dependent variable did not have homogeneity of variance. Neither predator nor the type of mesocosm had a significant effect on the proportion of crabs observed in corners (p>0.40; Table S8). The proportion of crabs observed along edges was significantly affected by predator (F_1,15_ = 6.37, p = 0.023; Fig. 2 D; Table S9) and mesocosm type (F_1,15_ = 5.54, p = 0.033). The interaction between these 2 factors was also significant (F_1,15_ = 5.95, p = 0.028). Neither predator nor the type of mesocosm had a significant effect alone on the proportion of crabs observed in the oyster habitat (p>0.40; Table S10), but the interactions between these two independent variables was marginally significant (F_1,15_ = 3.24, p = 0.088).

The contributions of predator-avoidance behaviors and consumption of prey on resource survival are summarized in Table 1. The presence of toadfish reduced the number of mussels eaten per crab per day by half. On average there was a small effect of mesocosm design and open mesocosms had 8% higher levels of per-prey consumption. The DMIE was almost 3-times greater in the closed (1.31±0.80) than in open mesocosms (0.46±0.46). This resulted from the significantly higher predation on mud crabs in closed mesocosms. The average number of crabs that dispersed out of an open mesocosm was the same for no-predator (1.33±0.21) and predator treatments (1.33±0.42). Although prey (crab) density remained unchanged in closed no-predator treatments, reduction in prey density resulting from predation and/or dispersal was similar between closed predator, open no-predator, and open predator treatments (1.33±0.42, 1.33±0.21, and 1.50±0.59 crabs respectively). The DR, or the number of mussels not eaten because of crab dispersal induced by predator presence, was −2.21±0.70. This negative number indicates that the DR (number of mussels “saved”) was lower in the presence of a predator than in the absence of a predator, an outcome that resulted from higher consumption of resources per prey in no-predator treatments. The activity resource release (AyR) was lower in the closed (4.19±2.06) than open (7.58±3.46) mesocosms. Finally, the activity (AyR) and dispersal (DR) resource release were combined for open mesocosms to calculate the number of resources not eaten resulting from both of these prey behaviors (5.37±3.13).

The contribution of TMIEs as compared to DMIEs was calculated for closed and open mesocosms. The TMIEs from activity reduction in the closed mesocosm accounted for 76.2% of the indirect effect. The TMIEs in the open mesocosm, or the increase in resource survival resulting from changes in prey foraging activity and dispersal, accounted for 92.1% of the effect of the predator on the resource. The difference in indirect effects between the treatments was primarily driven by the significantly higher predation on mud crabs in closed mesocosms.

## Discussion

Reduced foraging activity and dispersal of prey were both more important than consumption of prey in the indirect effect of a predator on resources. Our results add to the growing body of evidence that fear of predation can have a greater influence on food chain dynamics than predation. The evidence includes experiments in grass meadows [11], [43], [44], freshwater streams [27], [45]–[47], and intertidal pools [10], [48]. But, unlike these past studies, we separated the relative effect of multiple predator avoidance behaviors. We found that when prey were unable to disperse (closed mesocosms), TMIEs on mussel survival were 3 times higher than the DMIEs. Although the ability to disperse did not directly affect mussel survival, the indirect effects that resulted in mussel survival did change. When crabs were allowed to disperse, the TMIEs on mussel survival increased to 11 times the DMIEs. This increase in TMIEs resulted from rates of mud crab consumption by toadfish (the sole source of DMIEs) that were 9 times higher in closed mesocosms than in mesocosms where crabs could disperse. Crabs were observed moving along mesocosm edges more often in closed mesocosms than in open mesocosms, probably because they were trying to disperse. This left prey more vulnerable to predation and increased prey mortality and estimation of DMIEs. Open mesocosms had only 1 of 4 sides permeable to crabs, and yet prey consumption by a predator was significantly reduced as compared to closed mesocosms. Predation resource release (DMIEs) could be even lower in natural settings because no mesocosm boundaries exist, but this is dependent on predator density because prey could inadvertently move into an area with predators. Mesocosm experiments on indirect effects could be overestimating DMIE because of mesocosm artifacts, especially when mesocosm size restricts the distance prey can move in relatively short time periods (<1 minute), a limitation that is common in previous indirect effect experiments (Table S1). However, the magnitude of the potential bias resulting from mesocosms is context dependent and is probably affected by the predator-avoidance behaviors of the prey, the forage area of the prey and predator (home range), and whether the predator actively searches for prey or ambushes prey.

Prey can reduce predator encounters by dispersing away from a predator [19], [26], [28], [49], [50]. We found that the percent of surviving mud crabs remaining in the mesocosm was not affected by toadfish presence, which is supported by a smaller body of literature that shows no effect of predators on prey dispersal [25], [31], [51]. Crabs that remained in a predator mesocosm ate fewer mussels than crabs that remained in the predator-free mesocosms, which led to higher mussel survival in predator-free mesocosms because of crab dispersal. This resulted in a negative dispersal resource release because the predator had a negative effect on resource survival. The effect of dispersal on resource survival was four times greater in magnitude than the effect of prey mortality. Our results bring up interesting scenarios in which the cascading effect of dispersal is not intuitive, such as when a predator does not alter prey dispersal, but does decrease TMIEs. This could occur when the per-prey consumption of resources is lower in the presence of a predator. Or, a dispersal resource release could be negligible even though predators increased dispersal, because per-prey consumption decreased in the presence of a predator.

Unlike prey that reduce activity in the presence of a predator, prey that disperse probably affect resources in the area where the prey disperse to. This is known as ‘remote effects’ of predators [31] and is seldom quantified. We found that a predator has a disproportionately larger effect on resource survival in sanctuaries, where resource consumption was 5 times greater when there was no predator, as opposed to when there was a predator in the mesocosm. This was probably a consequence of both chronic predator effects [52], in which prey that were recently under threat of predation remain vigilant, and a consequence of prey continuing to detect the predator in the mesocosm (e.g. chemical and/or visual cues). Remote predator effects are not only dependent on whether prey alter dispersal rates in the presence of a predator, but also the distance from a predator in which the prey resume foraging without ‘fear’. Although such effects are dependent on the spatial scale, incorporating the effect of dispersing prey on the resource outside of the study area is important in understanding the overall effect of predator-avoidance behavior on resource populations.

The indirect effects of prey mortality and reduced prey activity were previously investigated in a tri-trophic food chain with toadfish, mud crabs, and juvenile oysters [22], [23]. Grabowski [22]found a TMIE that was larger than what we found in a closed mesocosm (TMIE was ≥94% compared to our finding of 76.2%). Several factors may explain these differences. First, the effect of a predator on avoidance behavior is dependent on prey density (all prey get scared regardless of their density; [8], [53]) and indirect effect calculations are based on the change in resource consumption by all the prey. Thus, indirect effects can change depending on the prey density, and if feasible, it is best to measure indirect effects as a function of prey density [20]. The prey to predator ratio that Grabowski [22] used was double ours, which probably resulted in a larger TMIE. But, consumption of mud crabs per toadfish in Grabowski’s study was similar to ours in closed mesocosms (0.5 vs. 0.6 crabs·day^−1^) but not in open mesocosms (0.06 crabs·day^−1^), which suggests that mesocosms used in Grabowski’s study could have overestimated the relative importance of DMIEs compared to more natural conditions. Our experiment had a patch of oyster habitat surrounded by open substrate, whereas Grabowski [22]had oyster reef covering the entire mesocosm. Many habitats, including oyster reefs, exist in a continuum of patch sizes and prey dispersing from habitat patches are often more vulnerable to predation [54]. Although patch size and configuration of habitat has not been included in indirect effect experiments, it too may alter indirect effects [55]. Habitat quality may have also influenced mud crab dispersal in our experiment. Grabowski [22]found that mud crabs are consumed by toadfish on low complexity reefs similar to those in this experiment (i.e., low relief dead shell), but were not at risk in high complexity reefs that mimicked intact reefs with high relief created by living oysters. Further research on the influence of patch size, landscape setting, prey density and refuge quality on the relative strength of TMIEs is needed to broaden our understanding of food web dynamics and ability to model these interactions.

While the limited spatial and temporal scales of indirect effect experiments are cited as reasons why the results may not be scalable to natural food webs [56], [57], the number of large-scale studies finding that predator-avoidance behaviors are just as important as prey mortality in indirect effects is growing [15], [58]–[61]. Animal behavior is at the interface between selection pressure and population dynamics [13], and thus integral to our ability to understand and predict changes in ecological communities. Our findings show that complex prey behavior is important in determining the effect of a predator on local resources, and ignoring particular predator-avoidance behaviors can overestimate the importance of predators consuming prey on indirect effects of predators.

## Supporting Information

Figure S1 **Oyster reef habitat at the Rachel Carson Research Reserve, NC (76°38.5′ Lon, 34°42.5′ Lat); (1) dispersed, (2) intermediate sized patches, and (3) continuous habitat.**
(TIF)

Table S1 **Summary of studies using 3 tropic levels in a meta-analysis by Preisser et al.** [**7**] **followed by references cited.** We estimated the time scale required for prey to traverse the mesocosm based on the natural history of the prey.
(DOCX)

Table S2 **Two-way ANOVA with mesocosm (open/closed) and trial as independent variables and proportion of crabs consumed per trial as the dependent variable.**
(DOCX)

Table S3 **Three-way ANOVA with toadfish (presence/absence), mesocosm (open/closed), and trial as independent variables and proportion of crabs remaining in the mesocosm as the dependent variable.**
(DOCX)

Table S4 **Two-way ANOVA with toadfish (presence/absence) and trial as independent variables and number of crabs that moved into the sanctuary as the dependent variable.**
(DOCX)

Table S5 **Three-way ANOVA with toadfish (presence/absence), mesocosm (open/closed), and trial as independent variables and percent mortality of mussels per day as the dependent variable.**
(DOCX)

Table S6 **Two-way ANOVA with toadfish (presence/absence) and trial as independent variables and percent mussel mortality per day in the sanctuary as the dependent variable.**
(DOCX)

Table S7 **Two-way MANOVA with toadfish (presence/absence) and mesocosm (open/closed) as independent variables and number of crabs observed in corners, along edges, and in oyster habitat as dependent variables.**
(DOCX)

Table S8 **Three-way ANOVA with toadfish (presence/absence), mesocosm (open/closed), and trial (blocked) as independent variables and number of crabs observed in corners as the dependent variable.**
(DOCX)

Table S9 **Three-way ANOVA with toadfish (presence/absence), mesocosm (open/closed), and trial (blocked) as independent variables and number of crabs observed along edges as the dependent variable.**
(DOCX)

Table S10 **Three-way ANOVA with toadfish (presence/absence), mesocosm (open/closed), and trial (blocked) as independent variables and number of crabs observed in oyster habitat as the dependent variable.**
(DOCX)
