# Supplementary figures and images for: Restricting Prey Dispersal Can Overestimate the Importance of Predation in Trophic Cascades
Source: PLoS One. 2013 Feb 7;8(2):e55100. doi: 10.1371/journal.pone.0055100 (PMC3567106; doi:10.1371/journal.pone.0055100)

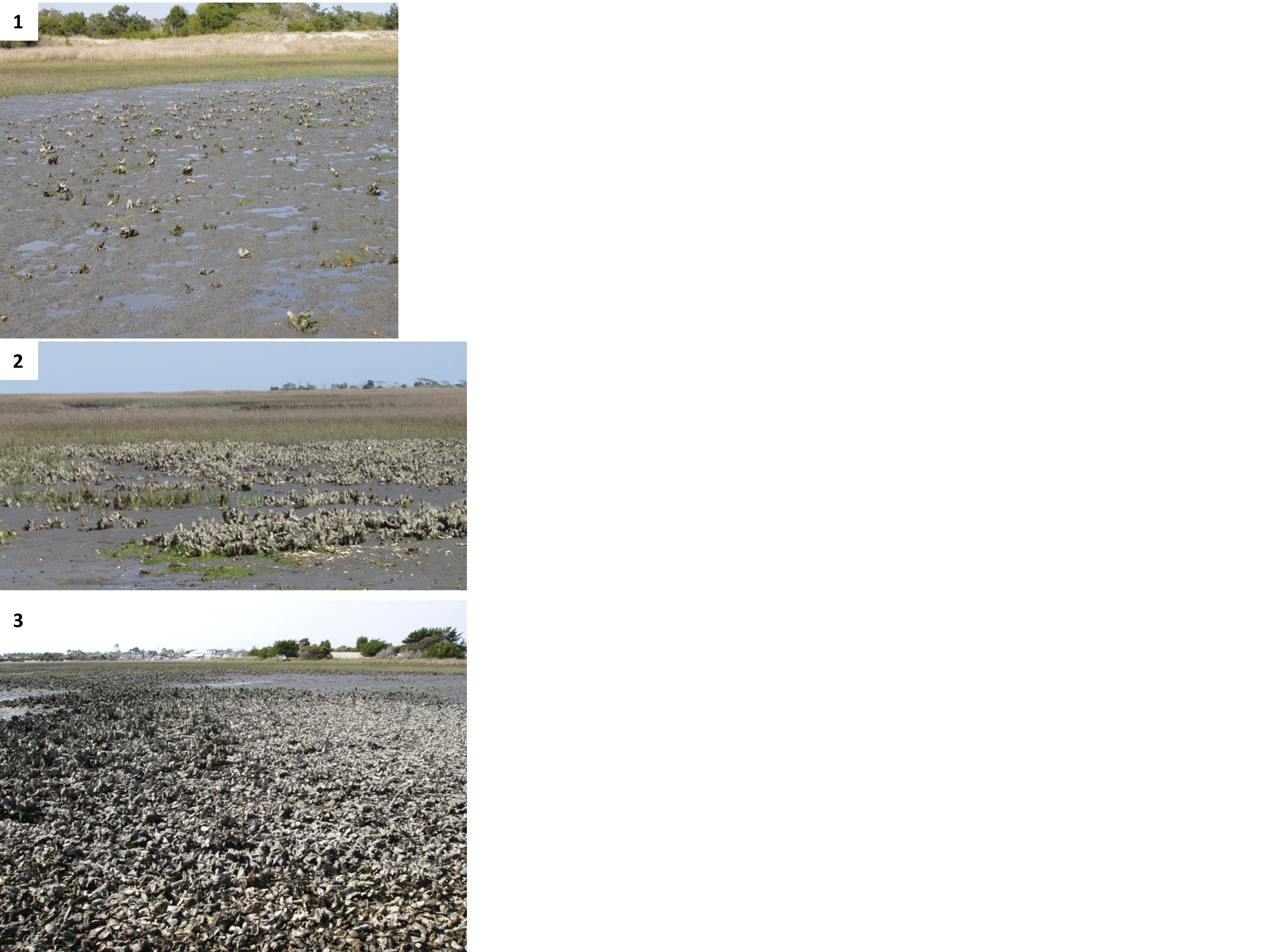

Supplement: Figure S1 — Oyster reef habitat at the Rachel Carson Research Reserve, NC (76°38.5′ Lon, 34°42.5′ Lat); (1) dispersed, (2) intermediate sized patches, and (3) continuous habitat. (TIF) [file pone.0055100.s001.tif]
