# Supplementary material for: Restricting Prey Dispersal Can Overestimate the Importance of Predation in Trophic Cascades
Source: PLoS One. 2013 Feb 7;8(2):e55100. doi: 10.1371/journal.pone.0055100 (PMC3567106; doi:10.1371/journal.pone.0055100)
Supplement: Table S2 — Two-way ANOVA with mesocosm (open/closed) and trial as independent variables and proportion of crabs consumed per trial as the dependent variable. (DOCX) [file pone.0055100.s003.docx]

**Table S2**.

| **Source of Variation** | **df** | **MS** | ***F*** | ***P*** |
| --- | --- | --- | --- | --- |
| Mesocosm | 1 | 0.276 | 6.636 | 0.030 |
| Trial | 5 | 0.038 | 0.924 | 0.362 |
| Residual | 5 | 0.42 |  |  |
