# Supplementary material for: Restricting Prey Dispersal Can Overestimate the Importance of Predation in Trophic Cascades
Source: PLoS One. 2013 Feb 7;8(2):e55100. doi: 10.1371/journal.pone.0055100 (PMC3567106; doi:10.1371/journal.pone.0055100)
Supplement: Table S3 — Three-way ANOVA with toadfish (presence/absence), mesocosm (open/closed), and trial as independent variables and proportion of crabs remaining in the mesocosm as the dependent variable. (DOCX) [file pone.0055100.s004.docx]

**Table S3**.

| **Source of Variation** | **df** | **MS** | ***F*** | ***P*** |
| --- | --- | --- | --- | --- |
| Predator | 1 | 14 | 0.03 | 0.857 |
| Mesocosm | 1 | 1530.7 | 3.66 | 0.075 |
| Trial | 5 | 267.3 | 0.64 | 0.674 |
| Predator x Mesocosm | 1 | 214 | 0.51 | 0.486 |
| Residual | 15 | 418.4 |  |  |
