# Supplementary material for: Restricting Prey Dispersal Can Overestimate the Importance of Predation in Trophic Cascades
Source: PLoS One. 2013 Feb 7;8(2):e55100. doi: 10.1371/journal.pone.0055100 (PMC3567106; doi:10.1371/journal.pone.0055100)
Supplement: Table S5 — Three-way ANOVA with toadfish (presence/absence), mesocosm (open/closed), and trial as independent variables and percent mortality of mussels per day as the dependent variable. (DOCX) [file pone.0055100.s006.docx]

**Table S5**.

| **Source of Variation** | **df** | **MS** | ***F*** | ***P*** |
| --- | --- | --- | --- | --- |
| Predator | 1 | 0.061 | 11.38 | 0.004 |
| Mesocosm | 1 | 0.003 | 0.5 | 0.49 |
| Trial | 5 | 0.014 | 2.64 | 0.066 |
| Predator x Mesocosm | 1 | 0.000 | 0.01 | 0.941 |
| Residual | 15 | 0.005 |  |  |
