# Supplementary material for: Restricting Prey Dispersal Can Overestimate the Importance of Predation in Trophic Cascades
Source: PLoS One. 2013 Feb 7;8(2):e55100. doi: 10.1371/journal.pone.0055100 (PMC3567106; doi:10.1371/journal.pone.0055100)
Supplement: Table S6 — Two-way ANOVA with toadfish (presence/absence) and trial as independent variables and percent mussel mortality per day in the sanctuary as the dependent variable. (DOCX) [file pone.0055100.s007.docx]

**Table S6**.

| **Source of Variation** | **df** | **MS** | ***F*** | ***P*** |
| --- | --- | --- | --- | --- |
| Predator | 1 | 0.040 | 4.32 | 0.092 |
| Trial | 5 | 0.025 | 2.64 | 0.156 |
| Residual | 5 | 0.009 |  |  |
