# Supplementary material for: Restricting Prey Dispersal Can Overestimate the Importance of Predation in Trophic Cascades
Source: PLoS One. 2013 Feb 7;8(2):e55100. doi: 10.1371/journal.pone.0055100 (PMC3567106; doi:10.1371/journal.pone.0055100)
Supplement: Table S7 — Two-way MANOVA with toadfish (presence/absence) and mesocosm (open/closed) as independent variables and number of crabs observed in corners, along edges, and in oyster habitat as dependent variables. (DOCX) [file pone.0055100.s008.docx]

**Table S7**.

| **Source of Variation** | **Df** | **Pillai** | ***approx. F*** | ***P*** |
| --- | --- | --- | --- | --- |
| Predator | 1 | 0.2890 | 2.445 | 0.097 |
| Mesocosm | 1 | 0.2700 | 2.218 | 0.121 |
| Predator x Mesocosm | 1 | 0.4040 | 4.059 | 0.023 |
| Residual | 20 |  |  |  |
