# Supplementary material for: Restricting Prey Dispersal Can Overestimate the Importance of Predation in Trophic Cascades
Source: PLoS One. 2013 Feb 7;8(2):e55100. doi: 10.1371/journal.pone.0055100 (PMC3567106; doi:10.1371/journal.pone.0055100)
Supplement: Table S9 — Three-way ANOVA with toadfish (presence/absence), mesocosm (open/closed), and trial (blocked) as independent variables and number of crabs observed along edges as the dependent variable. (DOCX) [file pone.0055100.s010.docx]

**Table S9**

| **Source of Variation** | **df** | **MS** | ***F*** | ***P*** |
| --- | --- | --- | --- | --- |
| Predator | 1 | 0.020 | 6.37 | 0.023 |
| Mesocosm | 1 | 0.017 | 5.54 | 0.033 |
| Trial | 5 | 0.005 | 1.6 | 0.219 |
| Predator x Mesocosm | 1 | 0.018 | 5.95 | 0.028 |
| Residual | 15 | 0.003 |  |  |
