# Supplementary material for: Restricting Prey Dispersal Can Overestimate the Importance of Predation in Trophic Cascades
Source: PLoS One. 2013 Feb 7;8(2):e55100. doi: 10.1371/journal.pone.0055100 (PMC3567106; doi:10.1371/journal.pone.0055100)
Supplement: Table S10 — Three-way ANOVA with toadfish (presence/absence), mesocosm (open/closed), and trial (blocked) as independent variables and number of crabs observed in oyster habitat as the dependent variable. (DOCX) [file pone.0055100.s011.docx]

**Table S10**

| **Source of Variation** | **df** | **MS** | ***F*** | ***P*** |
| --- | --- | --- | --- | --- |
| Predator | 1 | <0.001 | 0.562 | 0.463 |
| Mesocosm | 1 | <0.001 | 0.562 | 0.463 |
| Trial | 5 | <0.001 | 0.123 | 0.729 |
| Predator x Mesocosm | 1 | 0.002 | 3.240 | 0.088 |
| Residual | 15 | <0.001 |  |  |
